# Exploring the Effect of Lactium™ and *Zizyphus* Complex on Sleep Quality: A Double-Blind, Randomized Placebo-Controlled Trial

**DOI:** 10.3390/nu9020154

**Published:** 2017-02-17

**Authors:** Andrew Scholey, Sarah Benson, Amy Gibbs, Naomi Perry, Jerome Sarris, Greg Murray

**Affiliations:** 1 Centre for Human Psychopharmacology, Swinburne University of Technology, Hawthorn VIC 3122, Australia; sarahbenson@swin.edu.au (S.B.); Amygibbs57@gmail.com (A.G.); naomiperry21@gmail.com (N.P.); jsarris@unimelb.edu.au (J.S.); 2 ARCADIA Mental Health Research Group, The Professorial Unit, The Melbourne Clinic, Department of Psychiatry, Melbourne University, Richmond VIC 3121, Australia; 3 Psychological Sciences and Statistics, Swinburne University of Technology, Hawthorn VIC 3122, Australia; gwm@swin.edu.au

**Keywords:** lactium, *Zizyphus*, *Humulus lupulus*, nutritional supplements, complementary medicines, sleep disturbance, insomnia, LZComplex3, clinical trial

## Abstract

Acute, non-clinical insomnia is not uncommon. Sufferers commonly turn to short-term use of herbal supplements to alleviate the symptoms. This placebo-controlled, double-blind study investigated the efficacy of LZComplex3 (lactium™, *Zizyphus*, *Humulus lupulus*, magnesium and vitamin B6), in otherwise healthy adults with mild insomnia. After a 7-day single-blind placebo run-in, eligible volunteers (*n* = 171) were randomized (1:1) to receive daily treatment for 2 weeks with LZComplex3 or placebo. Results revealed that sleep quality measured by change in Pittsburgh Sleep Quality Index (PSQI) score improved in both the LZComplex3 and placebo groups. There were no significant between group differences between baseline and endpoint on the primary outcome. The majority of secondary outcomes, which included daytime functioning and physical fatigue, mood and anxiety, cognitive performance, and stress reactivity, showed similar improvements in the LZComplex3 and placebo groups. A similar proportion of participants reported adverse events (AEs) in both groups, with two of four treatment-related AEs in the LZComplex3 group resulting in permanent discontinuation. It currently cannot be concluded that administration of LZComplex3 for 2 weeks improves sleep quality, however, a marked placebo response (despite placebo run-in) and/or short duration of treatment may have masked a potential beneficial effect on sleep quality.

## 1. Introduction

Insomnia is defined by disturbances in sleep quality together with impairment of daytime functioning, for example fatigue and low mood [1]. Disturbances in sleep quality include difficulty getting to sleep, staying asleep or experiencing non-restorative sleep despite adequate opportunity for sleep [1]. An estimated 13%–33% of Australians experience some form of insomnia, similar to the estimated rates of insomnia in Western countries including Canada and the United States and in low-income countries across Africa and Asia [2,3,4]. Insomnia can occur as an acute episode, usually triggered by factors such as ill health, change of medication or circumstances, or stress [5]. Such sleep disturbances generally resolve without treatment once the trigger is eliminated. However, people can also turn to short-term use of medications (typically hypnotics such as a benzodiazepine) or herbal supplements during these episodes of insomnia [5,6,7]. In contrast, long-term or chronic insomnia can involve the development of maladaptive behaviors and a different treatment approach is required [5].

Commonly used herbal supplements for insomnia often include single or combined formulations of lemon balm (*Melissa officinalis*), chamomile (*Matricaria recutita*), valerian (*Valeriana* spp.), hops (*Humulus lupulus*), passionflower (*Passiflora incanata*), lactium™ (α_S1_-casein hydrolysate) and sour date (*Zizyphus jujube var. spinosa*) [8]. A new combined formulation, LZComplex3, contains lactium, sour date and hops, plus magnesium and vitamin B6 (pyridoxine) to provide nutritional support for metabolic pathways involved in sleep regulation. The rationale for the use of lactium as a sleeping aid originates from the observation that milk calms and soothes newborns [9]. The milk compound thought to be responsible for the calming or anxiolytic effects is a hydrolysate of α_S1_-casein, the bioactive peptide α-casozepine [10]. Lactium is the manufactured form of α_S1_-casein hydrolysate containing the α-casozepine peptide. Clinical studies have demonstrated that lactium reduces some symptoms related to stress [11,12]. Lactium has also been shown to have anxiolytic effects and to improve stress-induced sleep disturbance in animal studies [10,13,14].

Sour date (*Zizyphus jujube var. spinosa*; alternative spelling *Ziziphus*) is a fruit used in traditional Chinese medicine for its mild sedative and calming properties, to relieve irritability and aid sleep [15,16]. In combination with other herbs, it has been reported to improve mood and performance in individuals with anxiety and to improve sleep quality and a sense of well-being in individuals with sleep disorders [17,18]. Hops (*Humulus lupulus*) have been used in traditional western medicine for the treatment of mood disturbances such as restlessness and anxiety and in sleep disturbances due to reported calming and sleep-promoting properties [19,20,21,22,23,24]. Magnesium is involved in more than 300 metabolic reaction pathways including the production of melatonin, which regulates the sleep cycle [25]. Human and animal studies have implicated magnesium in the modulation of sleep [26,27,28,29,30,31]. Vitamin B6 may indirectly promote sleep quality through its role in the synthesis of a number of neurotransmitters involved in sleep regulation, including dopamine, serotonin, glutamate, *γ*-aminobutyric acid (GABA), and histamine [32,33]. 

Although the individual components in LZComplex3 have been studied with respect to their effects on sleep and/or stress (a common cause of sleeping difficulties), the efficacy of the combined formulation as a treatment for sleep disturbance has not been investigated. We report here the results of a clinical trial the primary objective of which was to investigate the short-term effect of LZComplex3 on sleep quality, mood and cognitive function in individuals with sleeping difficulties not caused by a primary sleeping disorder or other diagnosed condition.

## 2. Materials and Methods

### 2.1. Trial Design

**Figure 1:**
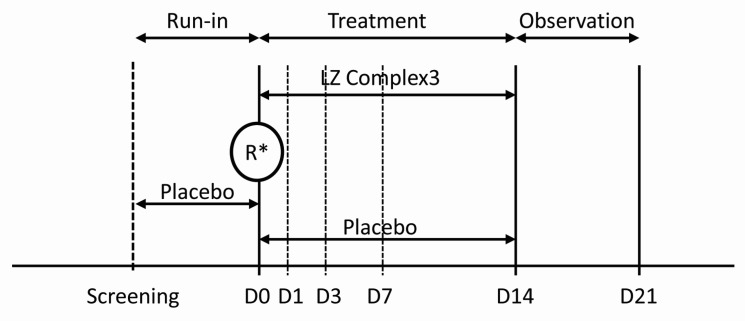
Study design. R* = randomization; D = study day.

This study was a placebo-controlled, double-blind, randomized, parallel group phase III trial with a single blind placebo run-in period (Figure 1). The trial was conducted at the Centre for Human Psychopharmacology, Swinburne University of Technology, Hawthorn, Victoria, Australia between 6 January 2014 and 23 December 2014. Ethical approval was granted by Bellberry Ltd., Eastwood, SA, Australia. The trial is registered with the Australian New Zealand Clinical Trials Registry (number ACTRN: 12613001363774) and was performed in accordance with the requirements for the conduct of clinical studies set by the Clinical Trial Notification (CTN) scheme of the Australian Therapeutic Goods Administration (TGA) and the Declaration of Helsinki.

Assessments of sleep quality, daytime functioning and physical fatigue, mood and anxiety, stress-reactivity, and cognitive function were completed by participants during the baseline and end of treatment visits, as well as during a final follow-up visit one week after the end of treatment. All assessment visits followed a procedure identical to that used at the baseline visit. In addition, participants completed all subjective sleep, daytime functioning, physical fatigue, mood and anxiety assessments at home 1, 3 and 7 days after baseline. All participant data were collected either at the study site (at screening, baseline, end of treatment, and final follow-up visits) or at the participants’ homes (interim assessments between baseline and end of treatment).

### 2.2. Study Participants and Randomization

Potentially eligible participants were identified in an initial telephone screen. Participant eligibility was confirmed at the initial screening visit; each eligible participant was allocated a unique participant number as soon as written informed consent was obtained and prior to any screening assessments. Mood questionnaires including the Hospital Anxiety and Depression Scale (HADS) [34] and State-Trait Anxiety Inventory Trait subscale (STAI-T) [35] were completed and participants were required to familiarize themselves with all the study assessments and procedures in order to reduce errors at baseline and practice effects. 

Eligible participants were healthy adults aged 18–65 years with no significant diagnosed diseases (as judged by the Investigator) who had self-reported sleeping difficulties over one month prior to the screening call. Following accepted practice [36], sleeping difficulties were defined as a Pittsburgh Sleep Quality Index (PSQI) Score >5 (PSQI scores range from 0 to 21 and higher scores indicate worse sleep quality). Participants with a primary sleep disorder (sleep apnoea-hypopnoea, periodic limb movement disorder, restless legs syndrome, narcolepsy, idiopathic hypersomnia, Kleine-Levin syndrome, as determined by subjective report and compilation of participants' medical history during the initial telephone screening assessment prior to randomization) were excluded. The full lists of inclusion and exclusion criteria are provided in Appendix A: Table A1. 

Eligible participants entered a one-week single-blind placebo run-in period, during which a daily sleep questionnaire (Consensus Sleep Diary; CSD [37]) was used to establish baseline sleeping criteria and detect placebo responders. Placebo response was based on CSD scores, and defined as sleep efficiency above 85%, sleep onset latency below 31 min, and wake after sleep onset below 31 min. 

At the baseline visit following the placebo run-in, placebo responders were excluded and all other participants were randomly assigned to treatment for two weeks (placebo or LZComplex3 in a 1:1 ratio) based on a randomization list generated centrally by an external independent third party. Participants and investigators were blinded to study treatment in the treatment phase and did not have access to the randomization codes except under exceptional medical circumstances.

Participants were invited at the baseline visit to wear an actiwatch to collect objective sleep data for exploratory cross-validation of the subjective sleep outcome measures. Participants who agreed were given an actiwatch to wear for the duration of the two-week treatment period.

### 2.3. Study Treatment

LZComplex3 tablets (Table 1) and placebo tablets were provided in blister packs and were matched for size, appearance, colour, smell and taste. The tablets were supplied to participants at the start of the placebo run-in and treatment phases in kit boxes. Each kit box contained sufficient blister packs of tablets to last for the duration of each phase of the trial plus an additional week to cover for any delays in attending the next scheduled visit. For the duration of each phase, participants were required to take two tablets daily, 30 min before retiring for sleep.

### 2.4. Primary and Secondary Outcome Measurments

The primary outcome was the change in overall sleep quality after two weeks of daily supplementation with LZComplex3. The primary outcome was measured by the change in PSQI scores from baseline to end of treatment at day 14. Secondary outcomes were the safety of LZComplex3 and the change in sleep quality, daytime functioning and physical fatigue, mood and anxiety, cognitive performance, and stress reactivity at 1, 3, 7 and 14 days after treatment with LZComplex3 and after one week post-treatment. The secondary outcomes were measured using validated assessments as outlined in Table 2. Objective measurement of sleep efficiency and time asleep using actigraph data from a subset of up to 90 participants was a pre-specified exploratory outcome designed to assess the use of actigraphy as a means of cross-validation of the primary and secondary endpoints. The Mini-Mitter Actiwatch-L (Respironics, Inc., Bend, Oregon) was used to collect actigraph data. Adverse Events (AEs), including Serious Adverse Events (SAEs) and Adverse Events of Special Interest (AESI), were collected at every visit. The AE observation period commenced the day of consent and finished at the final follow-up visit. 

#### 2.4.1. Screening Assessments

Screening assessments included the HADS, STAI-T, Leeds Sleep Evaluation Questionnaire (LSEQ), Bond-Lader Visual Analogue Scale (VAS), and the Stress and Fatigue Visual Analogue Mood Scales (VAMS). The HADS is a 14-item questionnaire designed to measure levels of anxiety and depression and was administered at screening to exclude participants with depression and/or anxiety [34]. The STAI-T comprises 20 different statements (e.g., “Some unimportant thought runs through my mind and bothers me”) [35]. Participants indicate how they generally feel on a scale ranging from “almost never” to “almost always”. Scores on the STAI-T range from 20 to 80, with higher scores indicating more anxiety. The Trait subscale of the STAI was to be used at screening to detect those participants who may have excessive levels of trait anxiety prior to commencing the study. 

#### 2.4.2. Treatment Assessments

Details of each assessment method are provided in Appendix A: Table A2.

### 2.5. Statistical Analyses

With an anticipated drop-out/non-compliance rate of 33%, it was estimated that a total of 170 participants would be required for 80% power to detect a medium effect size of approximately 0.5 at the 5% level of significance, with respect to the primary outcome. All analyses were conducted on a modified intention-to-treat (mITT) population, representing a per protocol/completer analysis and defined as all participants who were randomized and who had valid PSQI measures at both baseline and end of treatment. The primary outcome was also measured in the per protocol (PP) population, which included all participants in the mITT population who were at least 80% and less than 120% compliant with randomized treatment medication and had no major protocol deviations. Safety analyses were conducted on the safety population, which included all participants who were randomized and received at least one dose of study drug.

All measures were analyzed using SAS software (V9.4, SAS Statistical Institute, Cart, NC, USA). A general repeated measures mixed model was fitted to explore the difference between placebo and LZComplex3 in unadjusted change of total PSQI across all PSQI assessments for the mITT population. Day numbers and treatment group were included as fixed effects, participant as a random effect, the change in PSQI from baseline as the dependent variable and the baseline value of PSQI as a covariate. Secondary endpoints except ISI scores were analyzed in the same form as the primary endpoint. For the STAI-S, Bond-Lader VAS and VAMS scores, the mixed model also included time point (before and after administration of the MTF) as a fixed effect. A multinomial distribution and cumulative logit link function using PROC GLIMMIX was used to explore the difference between placebo and LZComplex3 in unadjusted change in ISI. The intended analysis of actigraphy data was not performed due to insufficient participant numbers (*n* = 16).

## 3. Results

### 3.1. Participant Characteristics

**Figure 2:**
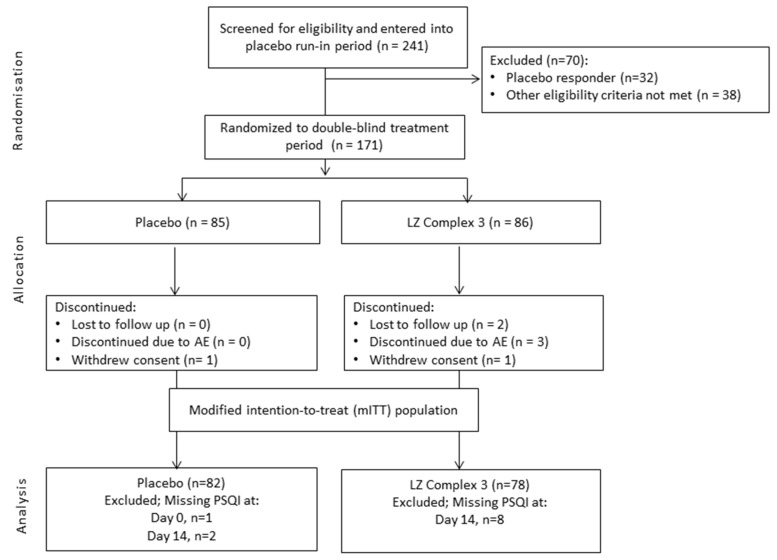
Participant flow. AE = adverse event, PSQI = Pittsburgh Sleep Quality Index

From a total of 241 participants, 171 were eligible for randomization following screening and the placebo run-in period. Eighty-five participants were allocated to the LZComplex3 group and 86 participants to the placebo group. After exclusions and losses, a total of 160 participants (LZComplex3, *n* = 78; placebo, *n* = 82) were eligible for analysis in the mITT population (Figure 2).

Participant demographics and other baseline characteristics are shown in Table 3. All characteristics were similar in the placebo and LZComplex3 groups. Most subjects were compliant during both the placebo run-in and treatment periods of the study. The mean compliance score, calculated as the percentage of study drug taken relative to the amount prescribed in the protocol, was 83.0 (standard deviation; SD 17.6) during the run-in period. The mean compliance scores in the LZComplex3 and placebo groups during the treatment period were 98.3 (SD 8.6) and 100.5 (SD 7.3) respectively.

### 3.2. Primary Outcome

**Figure 3:**
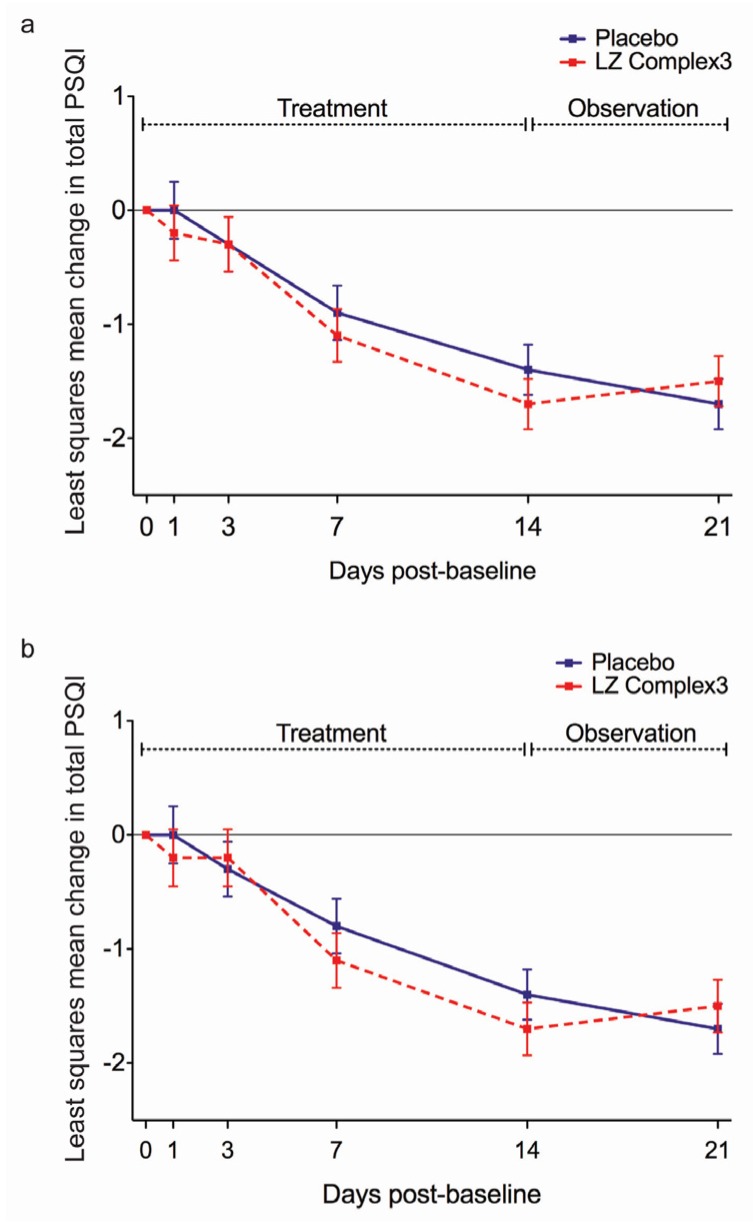
Least squares mean change in total Pittsburgh Sleep Quality Index (PSQI) between baseline (day 0) and end of the observation period (day 21) by treatment group in: (**a**) the modified intention-to-treat (mITT) population and (**b**) the per protocol (PP) population.

The mean change in total PSQI across time in the mITT population is presented in Figure 3a. Over the 2-week treatment period, the mITT population showed a gradual reduction in mean total PSQI from baseline to end of treatment (day 14) in both the LZComplex3 and placebo groups. Negative change scores indicate improved sleep quality in both groups, thus the change in both groups is in the direction of improved sleep. A mixed models analysis of covariance found a significant effect of day on PSQI (*F*_4,517_ = 30.40, *p* < 0.001) but no effect of treatment (*F*_1,157_ = 0.14, *p* = 0.713) and no interaction between treatment and day (*F*_4,517_ = 1.13, *p* = 0.340) in the mITT population. The results were similar in the PP population, with a significant effect of day (*F*_4,499_ = 29.63, *p* < 0.001), no effect of treatment (*F*_1,150_ = 0.17, *p* = 0.685) and no interaction between treatment and visit (*F*_4,499_ = 1.06, *p* = 0.374). The unadjusted PSQI scores between baseline and end of treatment are presented in Table 4. 

### 3.3. Secondary Outcomes

Similar to the primary outcome measure, the majority of secondary outcome measures showed similar changes in the placebo and LZComplex3 groups across the study period (Figures S1–S9, Table S1). A significant effect of day was found across all secondary outcomes measures except CSD domains, “Alertness” and ”Calmness” components of the Bond-Lader VAS, and ”Stress” and ”Fatigue” components of the VAMS. A cumulative logit link function in PROC GLIMMIX found a significant effect of treatment on ISI between baseline and end of the study. A between-group difference was detected in the proportions of participants with improved, worsened, or no change in ISI at day 3 compared with baseline (Figure S3). A mixed models analysis of covariance found a significant effect of treatment on STAI-S total scores that was due to a significant difference between the placebo and LZComplex3 groups in change in STAI-S total scores from pre- to post-administration of the MTF at day 0 and day 14 (Figure S7). There were no significant treatment effects detected across any other secondary outcome measures. Time point (before or after administration of the MTF) was found to have a significant independent effect on STAI-S total score, Bond-Lader VAS “Calmness” and “Contentedness” components, and VAMS “Stress” and “Fatigue” components (Figures S7–S9).

### 3.4. Safety

There were 25 AEs (19 mild, 5 moderate and 1 severe) reported during the run-in period, none of which were serious (defined as any experience which was fatal or life-threatening, was permanently disabling, required hospitalisation or prolongation of hospitalisation, was a congenital anomaly, or was an important medical event that could jeopardize the subject or require intervention to prevent one of those outcomes). Overall, 25 AEs were reported during the treatment period (Table 5). The proportion of participants reporting an AE was similar in each treatment group (placebo, *n* = 9 (10.6%; LZComplex3, *n* = 11 (12.9%); *p* = 0.8125; Table 5). The most common AEs were infections (placebo, *n* = 6; LZComplex3, *n* = 7) and gastrointestinal disorders (placebo, *n* = 0; LZComplex3, *n* = 4). There were no deaths or other serious adverse events. Two of the 4 AEs related to treatment in the LZComplex3 group led to permanent discontinuation. There were two hospitalizations reported, both in the LZComplex3 group, neither of which fulfilled the criteria of a serious AE.

## 4. Discussion

This study evaluated the efficacy of LZComplex3 in improving sleep quality in otherwise-well individuals with sleeping difficulties. There were no group differences in the primary outcome. Improvements in sleep quality were seen over a two-week treatment period with LZComplex3, however a persistent placebo response was observed and there was no significant treatment effect compared with placebo. Although the study included a one-week placebo run-in period designed to identify and exclude placebo-responders, it appears that that the run-in period may not have been of sufficient length. It is also possible that the persistent placebo response occurring after randomization may have been due to participants’ increased focus on overall sleep hygiene as a result of study visits and assessments, filling in a daily sleep diary and observing the protocol-mandated study parameters regarding stimulant use and sleep times. This degree of attention was not required in the placebo run-in phase. It has previously been suggested that having a patient keep a sleep diary for 2 weeks will aid with identification of behaviors that may worsen insomnia, thus providing a useful behavioral intervention [45]. Participants were required to fill in the CSD daily for the duration of the run-in and treatment periods, which may have contributed to the improvements in sleep quality observed in both treatment groups.

An improvement in sleep quality with LZComplex3 was expected, as previous studies have supported the use of individual components of the formulation as an aid for sleeping difficulties or insomnia (see Introduction). However, rigorous clinical studies are lacking. Effects of lactium on sleep quality have been investigated in a double-blind, controlled, parallel study of 32 Japanese patients experiencing poor sleep as determined by a global PSQI score greater than 4 [46]. As in the current study, improvements in sleep quality were observed over 4 weeks within the lactium group, however there were no significant differences between the placebo and lactium groups for any of the sleep components evaluated. It is possible that an effect of lactium was not detected due to a placebo response and the small sample size. Clinical evidence for the anxiolytic effects of lactium is more supportive. In a double-blind, randomized, controlled study of 42 healthy men treated with 3 doses each 12 h apart, experimental stress-induced elevations in blood pressure were significantly lower in the lactium group compared with the placebo group, supporting an anti-stress activity of lactium. As stress is a common cause of sleeping difficulties, it is thought that lactium may promote good quality sleep through its anti-stress activity.

The efficacy of sour date (*Zizyphus jujube var. spinosa*) in treating insomnia has been investigated in one clinical trial in the form of suanzaorentang [17,47], a popular Chinese herbal formula consisting of sour date (*Zizyphus jujube var. spinosa*), Fu Ling (mushroom) (*Poria cocos*), szechuan lovage (*Ligusticum wallichii*), Zhi Mu (*Anemarrhenae rhizoma*), and liquorice root (*Glycyrrhizae radix*) in a ratio of 7:5:2:1:1 [48]. The study compared self-rated measures of sleep quality in 60 participants with insomnia who received placebo for one week, followed by suanzaorentang for two weeks, followed by another week of placebo. All ratings of sleep quality significantly improved during the suanzaorentang treatment phase compared with the placebo periods. A number of trials evaluating the efficacy of suanzaorentang using benzodiazepines as the comparator showed favorable results for suanzaorentang in improving sleep, although the studies lacked methodological rigor [47].

Hops (*Humulus lupulus*) is considered to be a sedative agent, a view that originated from the observation of sleepiness in European hops-pickers [49]. It is commonly used in combination preparations with other herbs such as valerian (*Valeriana* spp.) and passionflower (*Passiflora incanata*) [50,51]. Although hops (*Humulus lupulus*) is listed as an approved herb for mood disturbances including sleep disturbances in The Complete German Commission E Monographs [52], there are no randomized controlled trials investigating the efficacy of hops (*Humulus lupulus*) alone in the treatment of insomnia. Thus it remains unclear whether hops (*Humulus lupulus*) has independent sedative effects, works as a synergist, or lacks sedative activity.

The rationale for inclusion of magnesium and vitamin B6 in LZComplex3 is based on in vitro and animal studies suggesting they may promote sleep quality by supporting metabolic pathways involved in sleep regulation, rather than due to any direct sedative activity [26,27,28,29,30,31,32,33]. Given the current clinical evidence base for the individual components in LZComplex3, it is difficult to determine whether the primary endpoint of this study was not met because of an absence of sedative activity, unknown complex pharmacokinetic interaction between the individual active ingredients, or because of methodological factors. 

It is possible that the two-week treatment duration in our study was not long enough to observe a treatment effect, and/or the one-week placebo run-in period was not long enough to eliminate all placebo responders. Another possibility is that the study population may have included individuals with chronic insomnia, which unlike brief or acute insomnia generally requires a cognitive behavioral approach to treatment [5]. The study population was selected based on a global PSQI score greater than 5, which indicates poor sleep [38]. However, individual’s responses on the PSQI questionnaire could not be used to differentiate between acute and chronic insomnia. Conversely, our ability to detect a treatment effect may be due to eligibility here being set at a minor level of sleeping difficulties, while individuals with more severe insomnia would be expected to benefit most from treatment.

Overall, the secondary outcome measures did not support a benefit of LZComplex3 over placebo during the two week treatment period. Participants completed a battery of questionnaires assessing various aspects of sleep quality, daytime functioning and physical fatigue, mood and anxiety, cognitive performance, and stress. Poor sleep can contribute to impairment of daytime functioning and physical fatigue thus these outcomes were anticipated to improve with better quality sleep. However, given that no significant difference between LZComplex3 and placebo was detected with respect to improvement in sleep quality as measured by the PSQI, it is not surprising that daytime functioning and physical fatigue were also not significantly different between the treatment groups. An improvement in ISI at day 3 favoring LZComplex3 and a between-group difference in stress reactivity as measured by change in STAI-S total score from pre- to post-administration of the MTF which appeared to be due to a slight increase in stress reactivity in the placebo group at baseline and at day 14 were the only significant treatment effects detected. Potential improvements in mood, anxiety and stress with LZComplex3 were anticipated as there is evidence that they may be positively impacted by some of the individual components of the formulation [10,11,12,13,14,17,18,19,20,21,22,23,24]. In our study, a clinically significant treatment effect may not have been detected for the same reasons as described for the primary outcome measure.

The safety data collected in this study indicate that LZComplex3 is well-tolerated at the dose of two tablets prior to sleep. Overall the safety profile of LZComplex3 was similar to placebo, with similar proportions of patients reporting AEs in both groups, the majority of which were mild or moderate and none of which were serious. There were more severe AEs reported in the LZComplex3 group and only two were considered possibly, probably or definitely related to treatment (both gastrointestinal disorders).

## 5. Conclusions

Despite finding a negative primary efficacy outcome, this study demonstrated an improvement in the PSQI and other measures of sleep quality for patients taking LZComplex3 with no deficits in cognitive or psychomotor function and a benign safety profile. These findings, taken into context with the marked placebo effect, short treatment duration and methodological limitations, suggest that further investigation of LZComplex3 is warranted.

## Figures and Tables

**Table 1:** LZComplex3 components.

Nutrient	Amount per Tablet
Lactium™ (hydrolysed milk protein; alpha casozepine enriched)	75 mg
Sour date (*Zizyphus jujube var. spinosa*) ext. equiv. to dry seed	4.5 g (4500 mg)
Hops (*Humulus lupulus*) ext. equiv. to dry flower	500 mg
Magnesium oxide (equivalent magnesium)	81.7 mg (52.5 mg)
Vitamin B6; pyridoxine hydrochloride (equivalent pyridoxine)	10 mg (8.23 mg)

**Table 2:** Outcome measures.

Outcome	Measurements
**Primary outcome**	
Sleep quality	Pittsburgh Sleep Quality Index (PSQI) [38]
**Secondary outcome**	
Sleep quality	Leeds Sleep Evaluation Questionnaire (LSEQ) [39]
	Epworth Sleepiness Scale (ESS) [40]
	Insomnia Severity Index (ISI) [41]
	Consensus Sleep Diary (CSD) [37]
Daytime functioning and physical fatigue	Burckhardt Quality of Life Scale (QoLS) [42]
	Chalder Fatigue Scale (CFS) [43]
Mood, anxiety and stress reactivity	Bond–Lader Visual Analogue Scales (Bond-Lader VAS) [44]
	State-Trait Anxiety Inventory (STAI) State subscale (STAI-S) [35]
	Stress and Fatigue Visual Analogue Mood Scales (VAMS)
Cognitive performance	Purple multi-tasking framework (MTF)

**Table 3:** Demographic and other baseline characteristics (safety population; *n* = 170).

Characteristic	Placebo (*n* = 85)	LZComplex3 (*n* = 85)
Gender		
Male, *n* (%)	38 (44.7)	36 (42.4)
Female, *n* (%)	47 (55.3)	49 (57.6)
Ethnicity		
Caucasian/White, *n* (%)	61 (71.8)	58 (68.2)
Black, *n* (%)	0 (0.0)	1 (1.2)
Asian/Oriental, *n* (%)	13 (15.3)	13 (15.3)
Other, *n* (%)	11 (12.9)	13 (15.3)
Age in years, mean (SD)	31.0 (10.5)	29.6 (9.05)
Height in cm, mean (SD)	172.6 (9.9)	171.2 (10.4)
Weight in kg, mean (SD)	71.3 (12.6)	70.0 (13.9)
Years of education, mean (SD)	16.6 (2.5)	17.0 (2.6)

SD = Standard deviation.

**Table 4:** Total PSQI, mean (SD).

	mITT Population			PP Population		
	*n*	Mean PSQI	Change from Baseline	*n*	Mean PSQI	Change from Baseline
**Baseline**						
Placebo	82	9.0 (2.5)		81	9.0 (2.1)	
LZComplex3	78	9.4 (2.6)		72	9.5 (2.6)	
**Day 1**						
Placebo	56	9.0 (2.3)	0.0 (2.1)	55	8.9 (2.3)	0.0 (2.1)
LZComplex3	61	9.2 (2.6)	−0.2 (1.3)	59	9.1 (2.6)	−0.3 (1.2)
**Day 3**						
Placebo	60	8.7 (2.3)	−0.4 (1.8)	59	8.7 (2.3)	−0.4 (1.8)
LZComplex3	60	9.2 (2.6)	−0.3 (1.4)	58	9.1 (2.6)	−0.3 (1.3)
**Day 7**						
Placebo	62	8.0 (2.4)	−0.9 (1.9)	61	8.0 (2.4)	−0.8 (1.9)
LZComplex3	67	8.2 (2.8)	−1.2 (2.1)	62	8.2 (2.7)	−1.2 (2.1)
**Day 14**						
Placebo	82	7.7 (2.7)	−1.3 (2.4)	81	7.7 (2.7)	−1.3 (2.5)
LZComplex3	78	7.6 (2.8)	−1.8 (2.1)	72	7.7 (2.8)	−1.8 (2.0)
**Day 21**						
Placebo	82	7.3 (2.5)	−1.7 (2.8)	81	7.3 (2.5)	−1.7 (2.8)
LZComplex3	77	7.9 (3.0)	−1.5 (2.3)	72	7.9 (3.0)	−1.6 (2.3)

mITT = modified intention-to-treat, PP = per protocol, PSQI = Pittsburgh Sleep Quality Index, SD = standard deviation.

**Table 5:** Summary of adverse events (AEs) reported during the treatment period (safety population).

Type	Placebo (*n* = 85)	LZComplex3 (*n* = 85)
AEs, *n*	11	14
Mild	5	5
Moderate	5	4
Severe	1	5
Patients reporting AEs, *n* (%)	9 (10.6)	11 (12.9)
Mild	5 (5.9)	5 (5.9)
Moderate	4 (4.7)	4 (4.7)
Severe	1 (1.2)	4 (4.7)
AEs leading to discontinuation, *n*	0	2
Abdominal pain	0 (0.0)	1 (1.2)
Dyspepsia	0 (0.0)	1 (1.2)
Serious AEs, *n*	0	0
AEs of special interest, *n*	0	0
AEs related to study treatment, *n*	0	4
Abdominal pain	0 (0.0)	1 (1.2)
Dyspepsia	0 (0.0)	1 (1.2)
Gastritis	0 (0.0)	1 (1.2)
Gastroenteritis	0 (0.0)	1 (1.2)

## Supplementary Materials

The following are available online at http://www.mdpi.com/2072-6643/9/2/154/s1, Figure S1: Adjusted mean change in Leeds Sleep Evaluation Questionnaire (LSEQ) scores, Figure S2: Adjusted mean change in Epworth Sleepiness Scale (ESS) scores, Figure S3: Adjusted mean change in Insomnia Severity Index (ISI), Figure S4: Adjusted mean change in Consensus Sleep Diary (CSD) scores, Figure S5: Adjusted mean change in the Chalder Fatigue Scale (CFS) total score, Figure S6: Adjusted mean change in the Burckhardt Quality of Life Scale (QoLS) total score, Figure S7: Adjusted mean change in the State-Trait Anxiety Inventory State subscale (STAI-S) score, Figure S8: Adjusted mean change in Bond-Lader Visual Analogue Scale (VAS) scores, Figure S9: Adjusted mean change in Visual Analogue Mood Scale (VAMS) scores, Table S1: Adjusted change in multi-tasking framework (MTF) total and component scores.

## Abbreviations

The following abbreviations are used in this manuscript:

AE	adverse event
CFS	Chalder Fatigue Scale
CSD	Consensus Sleep Diary
EOT	end of treatment
ESS	Epworth sleepiness scale
HADS	Hospital Anxiety And Depression Scale
ISI	Insomnia Severity Scale
LSEQ	Leeds Sleep Evaluation Questionnaire
mITT	Modified intention-to-treat
MTF	Multi-tasking framework
PP	per protocol
PSQI	Pittsburgh Sleep Quality Index
QoLS	Quality of Life Scale
STAI-S	State-Trait Anxiety Inventory State subscale
STAI-T	State-Trait Anxiety Inventory Trait subscale
VAMS	Visual Analogue Mood Scale

## Appendix A

**Table A1:** Participant inclusion and exclusion criteria.

**Inclusion Criteria**
Individuals (male and female) with no significant diagnosed diseases by the judgment of the Investigator, who self-report sleeping difficulties over one month prior to the screening call. Age 18–65 years. Body mass index 18–30 kg/m^2^. Normal vital signs (blood pressure less than 140/90 mmHg (i.e., systolic blood pressure less than 140 mmHg and a diastolic blood pressure less than 90 mmHg, and a heart rate between 60–100 bpm). Pittsburgh Sleep Quality Index Score >5. Typical bedtime between 9 p.m. and 12 a.m. Symptoms consistent with Primary Insomnia established at Screening. Signed written informed consent.
**Exclusion Criteria**
Hospital Anxiety And Depression Scale depression score >8 and/or anxiety score >12 (assessed at Screening) Regular use of illicit drugs, excessive or inappropriate use of over the counter or prescription drugs or excessive use of alcohol (assessed by the Investigator or delegate and/or reported by the volunteers). Smoking more than 10 cigarettes a day. Consumption of more than 10 cups of tea or coffee (or equivalent of other caffeine containing drinks) and/or consumption of these drinks after 5 p.m. Allergy to milk proteins, latex or LZComplex3 ingredients. Primary sleep disorder (sleep apnoea-hypopnoea, periodic limb movement disorder, restless legs syndrome, narcolepsy, idiopathic hypersomnia, Kleine-Levin syndrome). Use of medicinal products for sleep disorders (e.g., hypnotic agents, anxiolytics, herbal remedies, homeopathy for hypnotic purposes) in the month prior to inclusion, or exhibiting withdrawal symptoms from the use of medicinal products for sleep disorders at Screening. On-going non-pharmacological treatment of sleep disorders (e.g., cognitive behavioral therapy, relaxation therapy) Expected sleep disturbance from external sources during the study period (such as young children or other household disturbance). Previous failure on prescription sleep medication. Pregnancy or lactation. Current sleep disturbance due to pain or a general medical condition including but not limited to pain, cystitis, urinary frequency, heart burn or others by the judgment of the Investigator that would preclude participation in the study. Sleep Efficiency >85% AND Sleep Onset Latency below 31 min AND Wake after Sleep Onset below 31 min (assessed during week 1 using the Consensus Sleep Diary). Participants who withdraw consent during screening (participants who are not willing to continue or fail to return).

**Table A2:** Treatment assessments.

Sleep quality	The PSQI is a 19-item questionnaire that produces a global sleep quality score and the following seven component scores: sleep quality, sleep latency, sleep duration, habitual sleep efficiency, sleep disturbance, use of sleeping medications, and daytime dysfunction [38]. Scores range from 0 to 21 and are designed to differentiate between good and poor sleep quality. Higher score indicates worse sleep quality.
	The LSEQ is a standardised self-reporting instrument comprising ten 100 mm visual analogue scales that pertain to the ease of getting to sleep, quality of sleep, ease of awakening from sleep and alertness and behavior following wakefulness [39].
	The ESS is an 8-item questionnaire asking subjects to rate their probability of falling asleep on a scale of increasing probability from 0–3 for eight different situations that most people engage in during their daily lives, though not necessarily every day (e.g., sitting and reading, as a passenger in a car for an hour without a break) [40]. A score between 0 and 9 is considered to be normal while a score between 10 and 24 could indicate sleep problems.
	The ISI is a 7-item questionnaire that addresses the degree of distress or concern caused by different sleep problems including sleep-onset and sleep maintenance problems, satisfaction with current sleep pattern, interference with daily functioning, noticeability of impairment attributed to the sleep problem [41]. Total score ranges from 0 to 28, with a higher score suggesting more severe sleep problems.
	The CSD is a standardised sleep diary that measures sleep efficiency, wake after sleep onset, and sleep onset latency [37].
Daytime functioning and physical fatigue	The Burckhardt QoLS is a 16-item scale that measures six conceptual domains of quality of life: material and physical well-being; relationships with other people; social, community and civic activities; personal development and fulfilment; recreation, and independence, the ability to do for yourself [42]. Each domain is scored on a 7-point “delighted-terrible” scale, with a higher total quality of life score indicating better quality of life.
	The CFS is an 11-item self-rating measure of fatigue severity for both physical and mental symptoms. The total score is derived from the number of items in which the participant responded “Worse than usual” or “Much worse than usual”, with higher scores indicating greater fatigue, and negative change values indicating less fatigue [43].
Mood and anxiety	The Bond-Lader VAS comprises a total of 16 lines (approximately 100 mm) anchored at either end by antonyms (e.g., alert–drowsy, calm–excited), on which participants indicate their current subjective position [44]. Individual item scores are calculated as the percentage distance along the line. The Bond-Lader VAS was collapsed into three category scales of “alertness”, “contentedness”, and “calmness”.
	The STAI-S measures fluctuating levels of anxiety on a 20-item subscale [35]. Scores range from 20 to 80, with higher scores indicating more anxiety.
	Stress and Fatigue VAMS are single visual analogue scales aimed to gauge subjective mood experience at the present moment relating to stress and fatigue.
	The Bond-Lader VAS, STAI-S and Stress and Fatigue VAMS were each administered before and after the MTF in order to measure acute levels of anxiety in response to an experimental stressor.
Cognitive performance and stress reactivity	The purple MTF consists of two tasks assessing executive function (mathematical processing and stroop colour-word), a psychomotor task that has a set time limit for completion (target tracker), and a working memory task that presents stimuli at 10 s intervals (memory search), all conducted simultaneously. The MTF can be used to elicit acute psychological stress in laboratory settings to assess stress reactivity.

CFS = Chalder Fatigue Scale; CSD = Consensus Sleep Diary; ESS = Epworth Sleepiness Scale; ISS = Insomnia Severity Index; LSEQ = Leeds Sleep Evaluation Questionnaire; MTF = multi-tasking framework; PSQI = Pittsburgh Sleep Quality Index Score; QoLS = Quality of Life Scale; STAI-S = State-Trait Anxiety Inventory State subscale; VAMS = Visual Analogue Mood Scales; VAS = Visual Analogue Scales.
