# Supplementary material for: Exploring the Effect of Lactium™ and Zizyphus Complex on Sleep Quality: A Double-Blind, Randomized Placebo-Controlled Trial
Source: Nutrients. 2017 Feb 17;9(2):154. doi: 10.3390/nu9020154 (PMC5331585; doi:10.3390/nu9020154)
Supplement: Supplementary file 1 [file nutrients-09-00154-s001.docx]

Supplementary Materials: Exploring the Effect of Lactium™ and *Zizyphus* Complex on Sleep Quality: A Double-Blind, Randomized Placebo-Controlled Trial

Andrew Scholey ^1,^*, Sarah Benson ^1^, Amy Gibbs ^1^, Naomi Perry ^1^, Jerome Sarris ^1,2^ and
Greg Murray ^3^


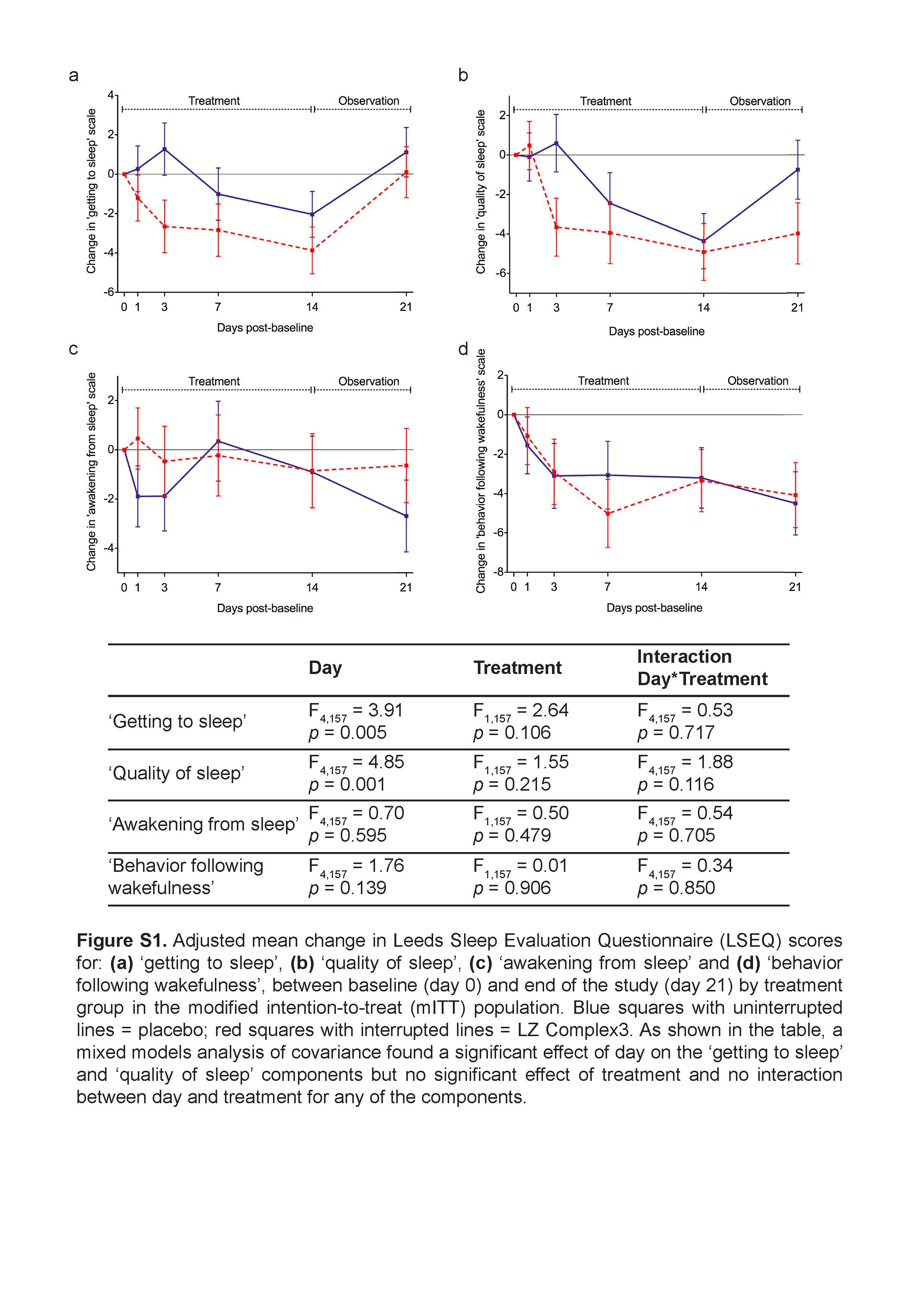


**Figure S1.** Adjusted mean change in Leeds Sleep Evaluation Questionnaire (LSEQ) scores for: **(a)** “getting to sleep”, **(b)** “quality of sleep”, **(c)** “awakening from sleep” and **(d)** “behavior following wakefulness”, between baseline (day 0) and end of the study (day 21) by treatment group in the modified intention-to-treat (mITT) population. Blue squares with uninterrupted lines = placebo; red squares with interrupted lines = LZComplex3. As shown in the table, a mixed models analysis of covariance found a significant effect of day on the “getting to sleep” and “quality of sleep” components but no significant effect of treatment and no interaction between day and treatment for any of the components.


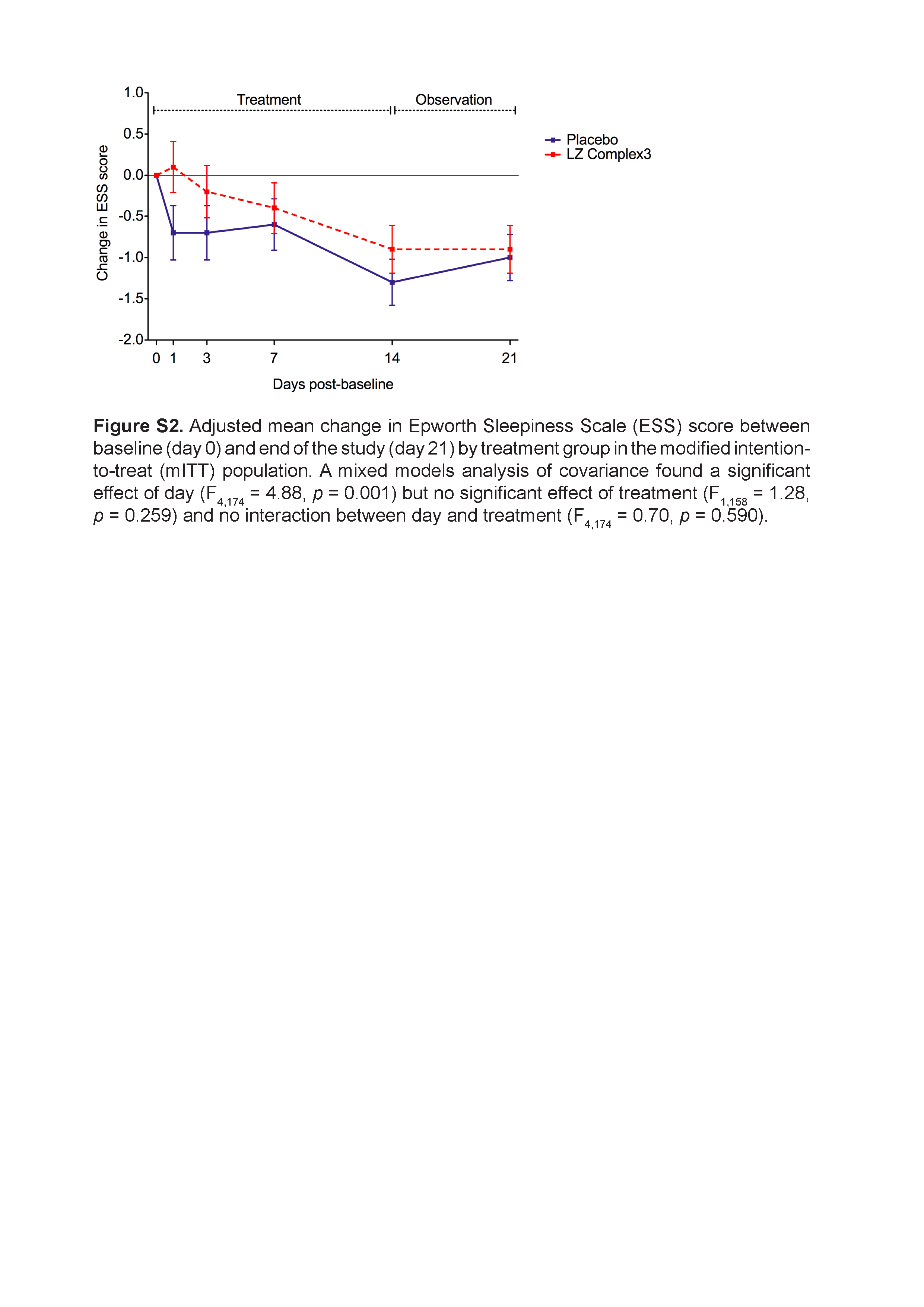


**Figure S2.** Adjusted mean change in Epworth Sleepiness Scale (ESS) score between baseline (day 0) and end of the study (day 21) by treatment group in the modified intentionto-treat (mITT) population. A mixed models analysis of covariance found a significant effect of day (*F*4,174 = 4.88, *p* = 0.001) but no significant effect of treatment (*F*1,158 = 1.28, *p* = 0.259) and no interaction between day and treatment (F4,174 = 0.70, *p* = 0.590).


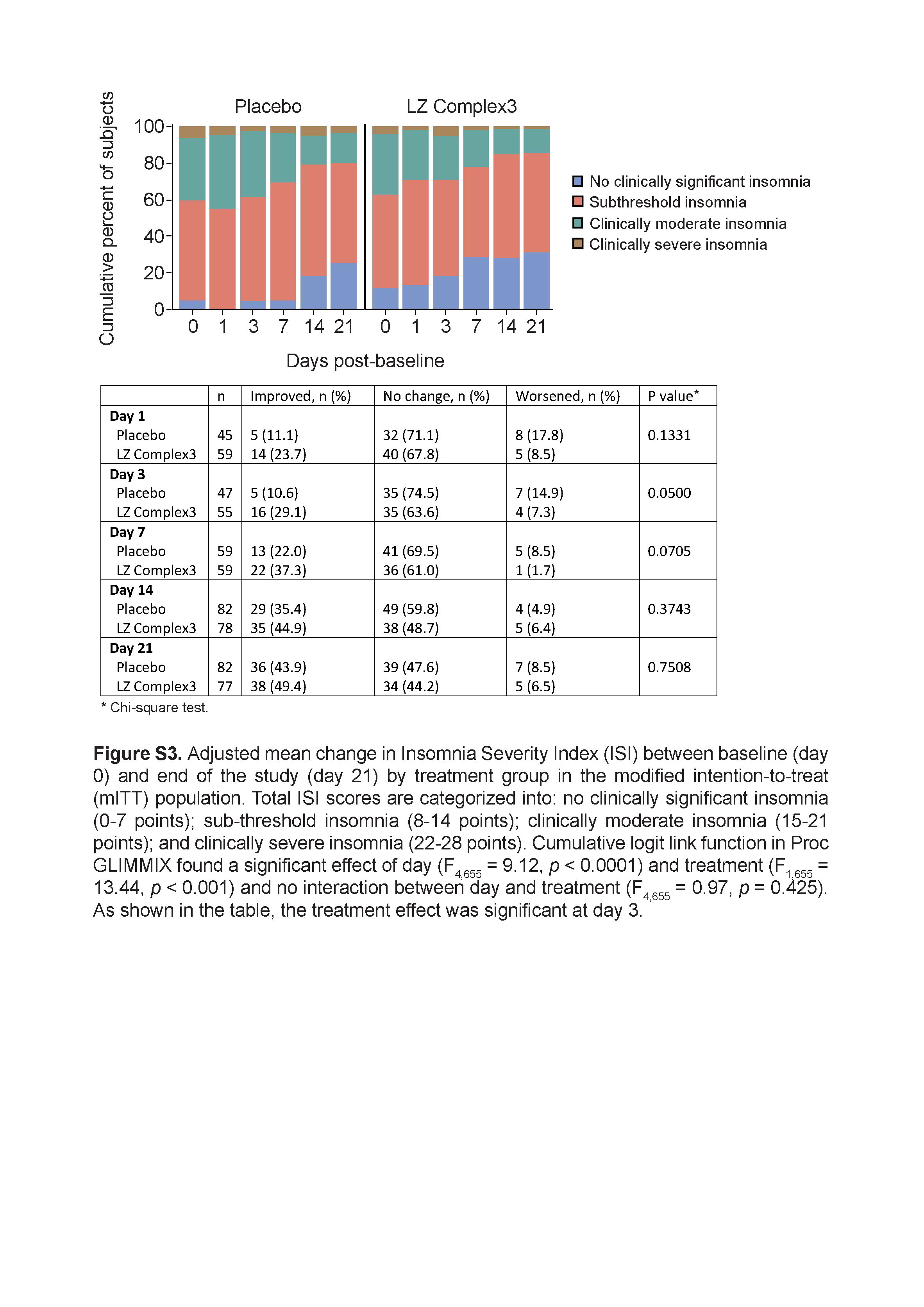


**Figure S3.** Adjusted mean change in Insomnia Severity Index (ISI) between baseline (day 0) and end of the study (day 21) by treatment group in the modified intention-to-treat (mITT) population. Total ISI scores are categorized into: no clinically significant insomnia (0-7 points); sub-threshold insomnia (8–14 points); clinically moderate insomnia (15–21 points); and clinically severe insomnia (22–28 points). Cumulative logit link function in Proc GLIMMIX found a significant effect of day (*F*4,655 = 9.12, *p* < 0.0001) and treatment (*F*1,655 =13.44, *p* < 0.001) and no interaction between day and treatment (*F*4,655 = 0.97, *p* = 0.425). As shown in the table, the treatment effect was significant at day 3.


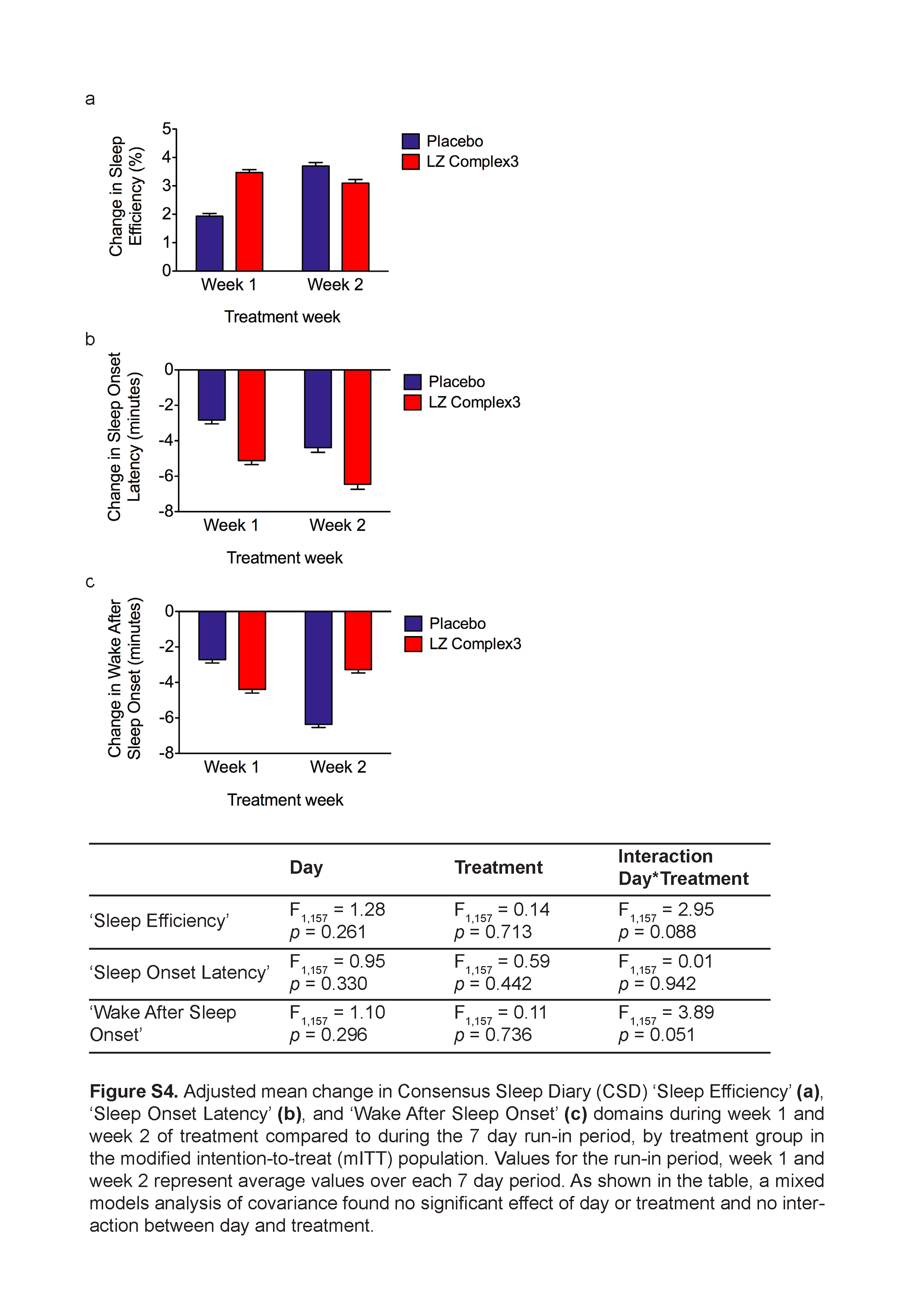


**Figure S4.** Adjusted mean change in Consensus Sleep Diary (CSD) “Sleep Efficiency” **(a)**, “Sleep Onset Latency” **(b)**, and “Wake After Sleep Onset” **(c)** domains during week 1 and week 2 of treatment compared to during the 7 day run-in period, by treatment group in the modified intention-to-treat (mITT) population. Values for the run-in period, week 1 and week 2 represent average values over each 7 day period. As shown in the table, a mixed models analysis of covariance found no significant effect of day or treatment and no interaction between day and treatment.


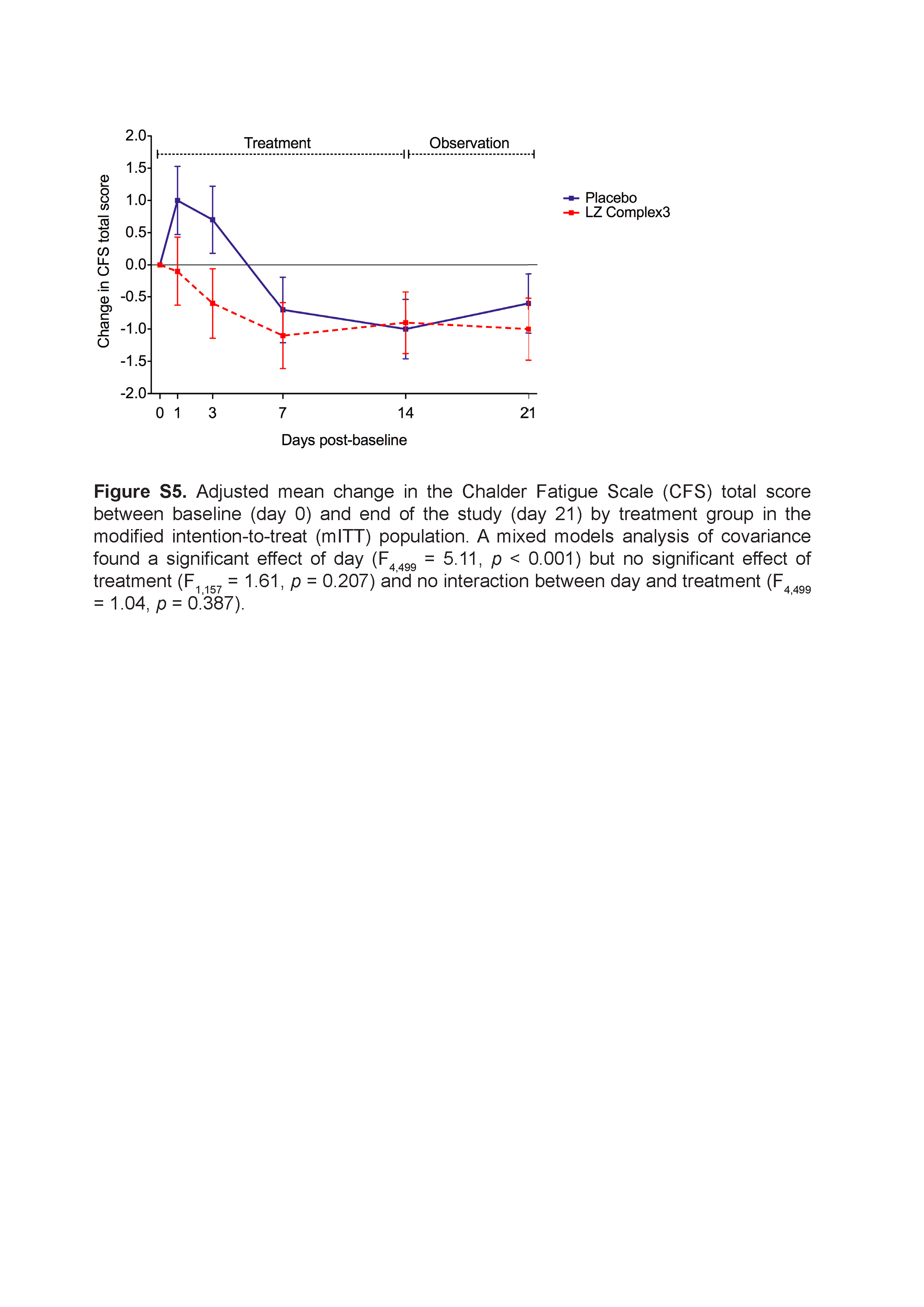


**Figure S5.** Adjusted mean change in the Chalder Fatigue Scale (CFS) total score between baseline (day 0) and end of the study (day 21) by treatment group in the modified intention-to-treat (mITT) population. A mixed models analysis of covariance found a significant effect of day (*F*4,499 = 5.11, *p* < 0.001) but no significant effect of treatment (*F*1,157 = 1.61, *p* = 0.207) and no interaction between day and treatment (*F*4,499 = 1.04, *p* = 0.387).


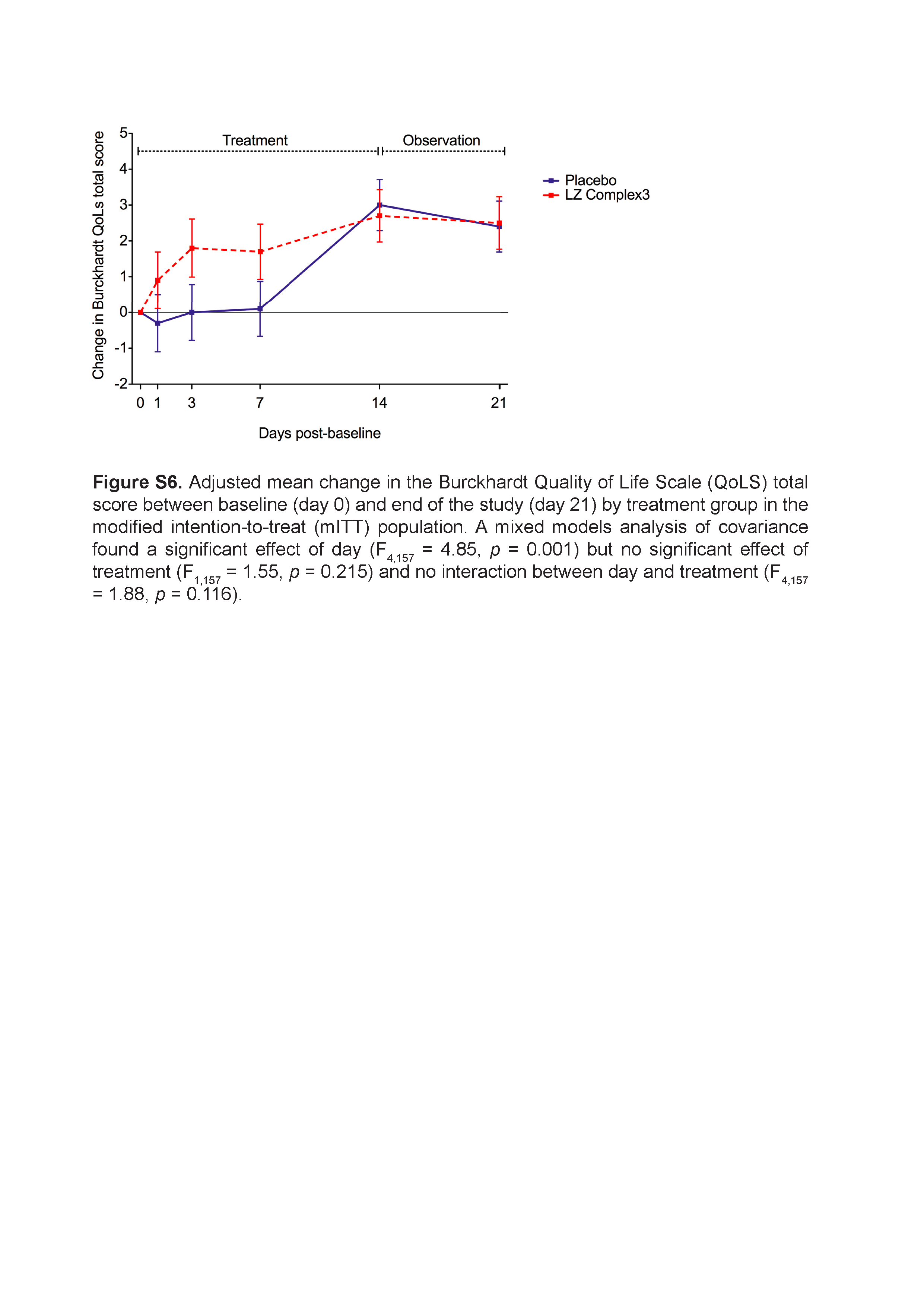


**Figure S6.** Adjusted mean change in the Burckhardt Quality of Life Scale (QoLS) total score between baseline (day 0) and end of the study (day 21) by treatment group in the modified intention-to-treat (mITT) population. A mixed models analysis of covariance found a significant effect of day (*F*4,157 = 4.85, *p* = 0.001) but no significant effect of treatment (*F*1,157 = 1.55, *p* = 0.215) and no interaction between day and treatment (*F*4,157 = 1.88, *p* = 0.116).


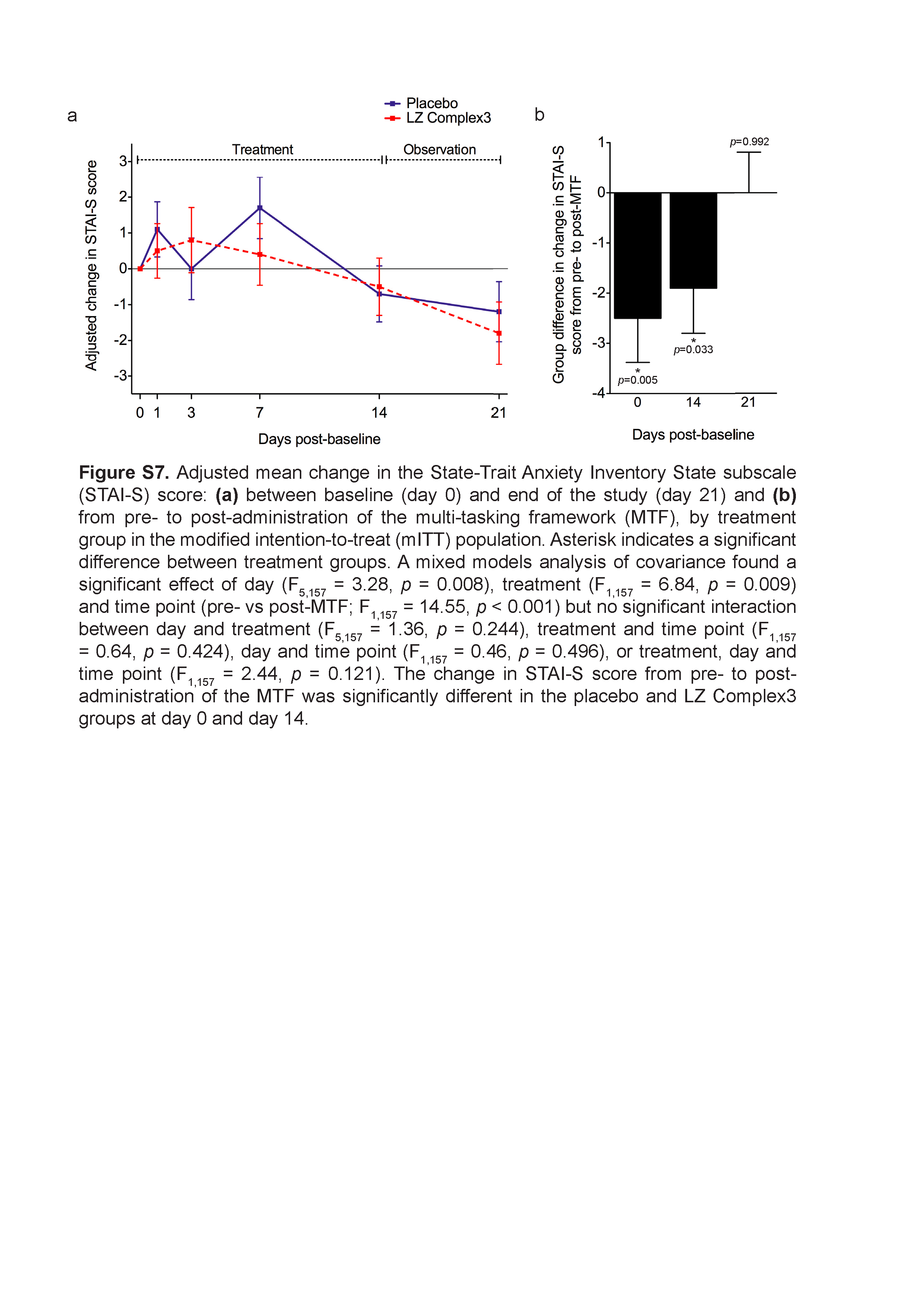


**Figure S7.** Adjusted mean change in the State-Trait Anxiety Inventory State subscale (STAI-S) score: **(a)** between baseline (day 0) and end of the study (day 21) and **(b)** from pre- to post-administration of the multi-tasking framework (MTF), by treatment group in the modified intention-to-treat (mITT) population. Asterisk indicates a significant difference between treatment groups. A mixed models analysis of covariance found a significant effect of day (*F*5,157 = 3.28, *p* = 0.008), treatment (*F*1,157 = 6.84, *p* = 0.009) and time point (pre- vs post-MTF; *F*1,157 = 14.55, *p* < 0.001) but no significant interaction between day and treatment (*F*5,157 = 1.36, *p* = 0.244), treatment and time point (*F*1,157 = 0.64, *p* = 0.424), day and time point (*F*1,157 = 0.46, *p* = 0.496), or treatment, day and time point (*F*1,157 = 2.44, *p* = 0.121). The change in STAI-S score from pre- to post-administration of the MTF was significantly different in the placebo and LZComplex3 groups at day 0 and day 14.


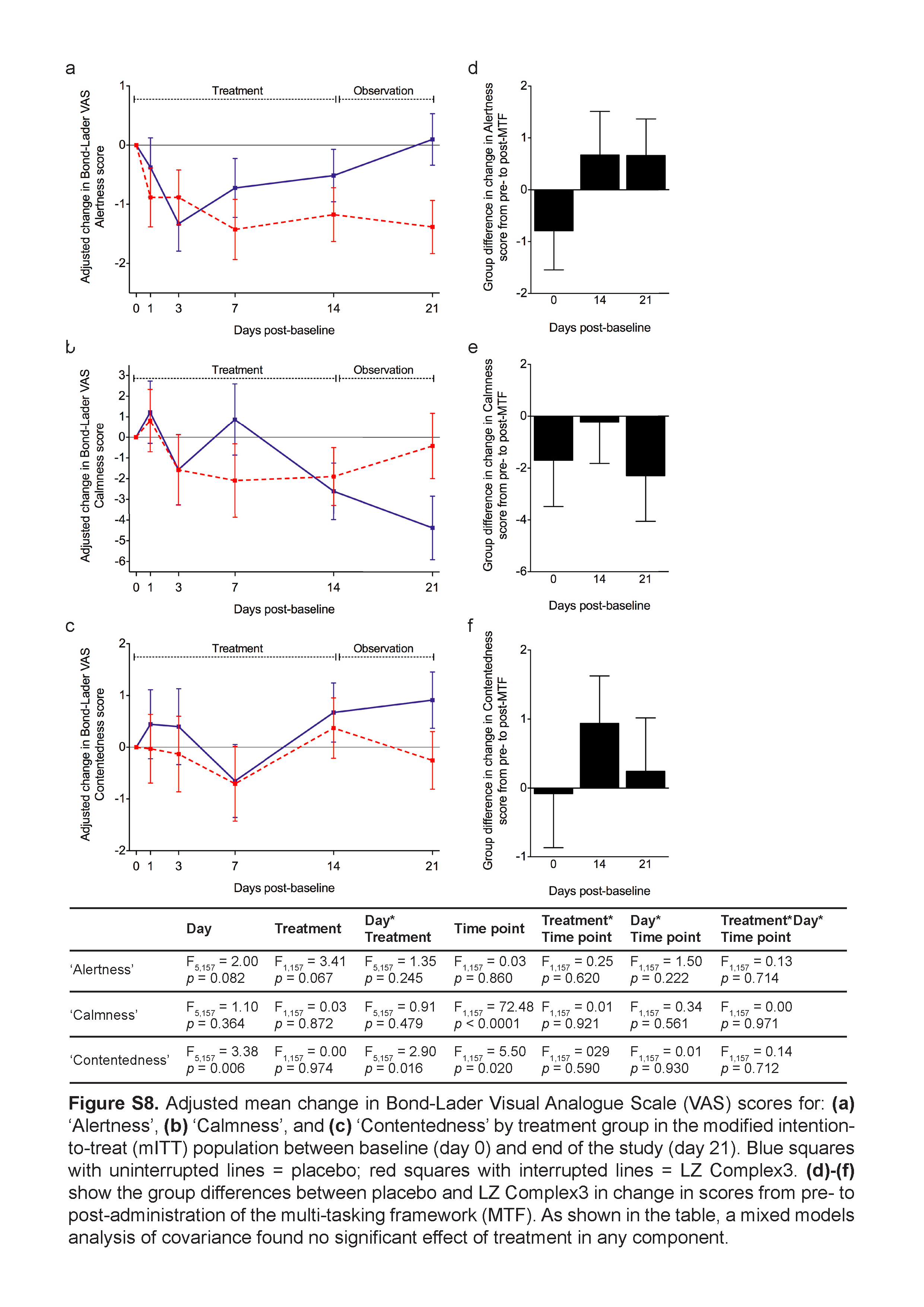


**Figure S8.** Adjusted mean change in Bond-Lader Visual Analogue Scale (VAS) scores for: **(a)** “Alertness”, **(b)** “Calmness”, and **(c)** “Contentedness” by treatment group in the modified intentionto-treat (mITT) population between baseline (day 0) and end of the study (day 21). Blue squares with uninterrupted lines = placebo; red squares with interrupted lines = LZComplex3. **(d**)–**(f)** show the group differences between placebo and LZComplex3 in change in scores from pre- to post-administration of the multi-tasking framework (MTF). As shown in the table, a mixed models analysis of covariance found no significant effect of treatment in any component.


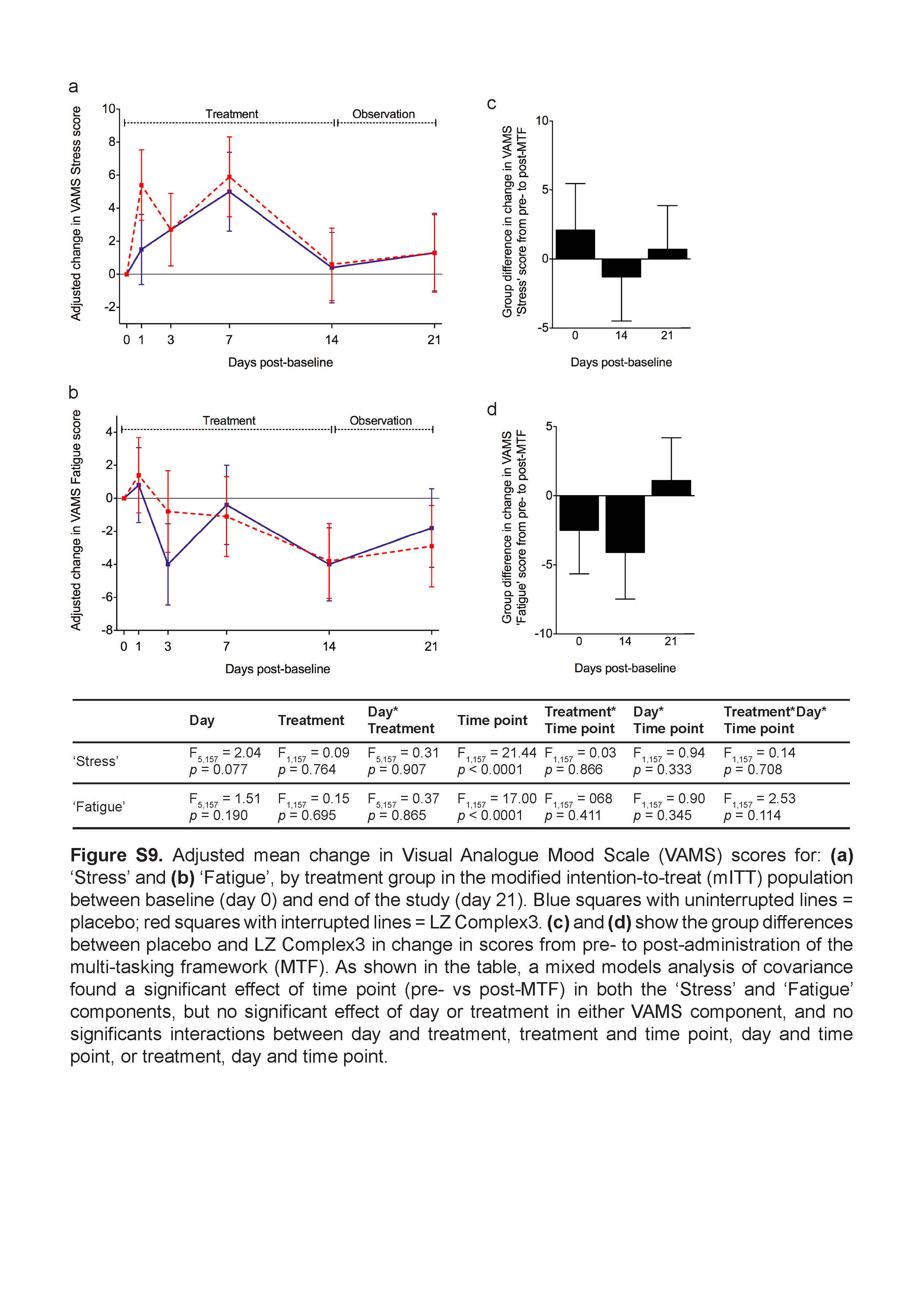


**Figure S9.** Adjusted mean change in Visual Analogue Mood Scale (VAMS) scores for: **(a)** “Stress” and **(b)** “Fatigue”, by treatment group in the modified intention-to-treat (mITT) population between baseline (day 0) and end of the study (day 21). Blue squares with uninterrupted lines = placebo; red squares with interrupted lines = LZComplex3. **(c)** and **(d)** show the group differences between placebo and LZComplex3 in change in scores from pre- to post-administration of the multi-tasking framework (MTF). As shown in the table, a mixed models analysis of covariance found a significant effect of time point (pre- vs post-MTF) in both the “Stress” and “Fatigue” components, but no significant effect of day or treatment in either VAMS component, and no significant interactions between day and treatment, treatment and time point, day and time point, or treatment, day and time point.

**Table S1.** Adjusted change in multi-tasking framework (MTF) total and component scores from baseline in the modified intention-to-treat (mITT) population.

|  |  | **Placebo (mean ± SD)** | **LZComplex3 (mean ± SD)** | **Difference between groups (mean ± SD)** | ***P* value** |
| --- | --- | --- | --- | --- | --- |
| **Total score** | **Day 14** | **986.1 ± 185.3** | **921.6 ± 190.0** | **−64.4 ± 265.8** | 0.8087 |
|  | **Day 21** | **1690.5 ± 241.9** | **1529.3 ± 250.1** | **−161.2 ± 348.3** | 0.6441 |
| **Math score** | **Day 14** | **−28.2 ± 13.7** | **2.5 ± 14.0** | **30.7 ± 19.6** | 0.1192 |
|  | **Day 21** | **−23.9 ± 16.8** | **0.6 ± 17.3** | **24.5 ± 24.1** | 0.3114 |
| **Math speed** | **Day 14** | **−644.3 ± 414.5** | **−448.5 ± 425.1** | **195.8 ± 594.4** | 0.7422 |
|  | **Day 21** | **−688.9 ± 598.2** | **−692.6 ± 616.9** | **−3.8 ± 859.7** | 0.9965 |
| **Math accuracy** | **Day 14** | **−1.7 ± 1.3** | **−0.2 ± 1.4** | **1.5 ± 1.9** | 0.4361 |
|  | **Day 21** | **−0.1 ± 1.2** | **−0.2 ± 1.3** | **−0. 2 ± 1.7** | 0.9256 |
| **Memory score** | **Day 14** | **306.1 ± 115.1** | **220.5 ± 118.1** | **−85.6 ± 165.2** | 0.6050 |
|  | **Day 21** | **291.5 ± 131.7** | **424.8 ± 136.5** | **133.3 ± 189.9** | 0.4839 |
| **Memory speed** | **Day 14** | **−242.7 ± 76.6** | **−194.1 ± 78.6** | **48.5 ± 109.8** | 0.6590 |
|  | **Day 21** | **−350.6 ± 75.8** | **−314.5 ± 78.4** | **36.1 ± 109.1** | 0.7409 |
| **Memory accuracy** | **Day 14** | **0.6 ± 0.3** | **0.2 ± 0.4** | **−0.4 ± 0.5** | 0.4158 |
|  | **Day 21** | **0.4 ± 0.4** | **0.3 ± 0.4** | **−0.1 ± 0.5** | 0.8343 |
| **Tracking score** | **Day 14** | **65.9 ± 12.1** | **41.1 ± 12.4** | **−24.8 ± 17.4** | 0.1564 |
|  | **Day 21** | **36.0 ± 19.1** | **55.9 ± 19.8** | **19.9 ± 27.5** | 0.4700 |
| **Tracking time** | **Day 14** | **−60.6 ± 9.1** | **−49.4 ± 9.3** | **11.2 ± 13.1** | 0.3938 |
|  | **Day 21** | **−32.3 ± 16.6** | **−62.5 ± 17.3** | **−30.2 ± 24.0** | 0.2095 |
| **Stroop score** | **Day 14** | **615.6 ± 189.8** | **685.5 ± 194.8** | **69.9 ± 273.4** | 0.7986 |
|  | **Day 21** | **1360.7 ± 277.7** | **1075.2 ± 288.0** | **−285.4 ± 401.0** | 0.4777 |
| **Stroop speed** | **Day 14** | **−93.4 ± 63.1** | **−132.6 ± 64.7** | **−39.2 ± 90.4** | 0.6656 |
|  | **Day 21** | **−172.5 ± 59.6** | **−164.9 ± 61.7** | **7.7 ± 85.9** | 0.9291 |
| **Stroop accuracy** | **Day 14** | **−0.6 ± 0.3** | **−0.1 ± 0.3** | **0.4 ± 0.5** | 0.3607 |
|  | **Day 21** | **−0.9 ± 0.4** | **−0.4 ± 0.4** | **0.5 ± 0.6** | 0.3788 |

SD = standard deviation.
